# Population genetic variations of the matrix metalloproteinases-3 gene revealed hypoxia adaptation in domesticated yaks (*Bos grunniens*)

**DOI:** 10.5713/ajas.17.0706

**Published:** 2018-10-29

**Authors:** Xuezhi Ding, Chao Yang, Pengjia Bao, Xiaoyun Wu, Jie Pei, Ping Yan, Xian Guo

**Affiliations:** 1 Key Laboratory of Yak Breeding Engineering, Lanzhou Institute of Husbandry and Pharmaceutical Sciences, Chinese Academy of Agricultural Sciences, Lanzhou 730050, China; 2 CAS Key Laboratory for Agro-Ecological Processes in Subtropical Region, National Engineering Laboratory for Pollution Control and Waste Utilization in Livestock and Poultry Production, South-Central Experimental Station of Animal Nutrition and Feed Science in Ministry of Agriculture, Hunan Provincial Engineering Research Center for Healthy Livestock and Poultry Production Institute of Subtropical Agriculture, The Chinese Academy of Sciences, Changsha 410125, China

**Keywords:** Yak, Matrix Metalloproteinases-3 (*MMP3*), Polymorphism, Hypoxia Adaptation

## Abstract

**Objective:**

As an iconic symbol of Qinghai-Tibetan Plateau and of high altitude, yak are subjected to hypoxic conditions that challenge aerobic metabolism. Matrix metalloproteinases-3 (*MMP3*) is assumed to be a key target gene of hypoxia-inducible factor-1α that function as a master regulator of the cellular response to hypoxia. Therefore, the aim of this investigation was to identify the DNA polymorphism of *MMP3* gene in domestic yak and to explore its possible association with high-altitude adaptation.

**Methods:**

The single-nucleotide polymorphisms (SNPs) genotyping and mutations scanning at the *MMP3* locus were conducted in total of 344 individuals from four domestic Chinese yak breeds resident at different altitudes on the Qinghai-Tibetan Plateau, using high-resolution melting analysis and DNA sequencing techniques.

**Results:**

The novel of SNPs rs2381 A→G and rs4331 C→G were identified in intron V and intron VII of *MMP3*, respectively. Frequencies of the GG genotype and the G allele of SNP rs2381 A→G observed in high-altitude Pali yak were significantly higher than that of the other yak breeds resident at middle or low altitude (p<0.01). No significant difference was mapped for SNP rs4331 C→G in the yak population (p>0.05). Haplotype GC was the dominant among the 4 yak breeds, and Pearson correlation analysis showed that the frequencies of GC was significantly lower in Ganan (GN), Datong (DT), and Tianzhu white yaks (TZ) compared with Pali (PL) yak. The two SNPs were in moderate linkage disequilibrium in high-altitude yaks (PL) but not in middle-altitude (GN, DT) and low-altitude (TZ) yaks.

**Conclusion:**

These results indicate that *MMP3* may have been subjected to positive selection in yak, especially that the SNP rs2381 A→G mutation and GC haplotypes might contribute to adaptation for yak in high-altitude environments.

## INTRODUCTION

Hypoxia is one of the most important factors affecting survival in high-altitude regions. Yak (*Bos grunniens*), a herbivore that differentiated from cattle (*Bos taurus*) about 4.4 to 5.3 million years ago [1], exclusively inhabit the Hindu Kush-Himalayan region and the Qinghai-Tibetan Plateau (QTP), at altitudes ranging from 3,000 to 5,500 m above sea level. Yaks are a source of livelihood for the Tibetan nomads and are integrally linked with the culture, religion and social life of its herders. In order to adapt to the harsh environment with lower partial oxygen pressure [2], strong ultraviolet (UV) radiation, and poor forage resources [3], the aboriginal animals have acquired enhancements to their metabolic capacity to maintain a physiological and genetic adaptations for persistence at high elevations that compensate for hypoxia [4,5].

The genome sequences data from most of amphibians and mammals have identified several important pathways and functional categories, including energy metabolism and oxygen transmission, response to hypoxia, DNA repair and ATPase production [6–8]. In particular, the mechanisms underlying plateau adaptability have been explored using population surveys of single-nucleotide polymorphism (SNP) data, successfully identifying candidate genes for genetic adaptation to the Tibetan Plateau [9]. Variations in endothelial PAS domain-containing protein 1 (*EPAS1*) including the other hypoxia-related genes were identified in the Tibetan Mastiff [10,11], Tibetan antelopes and Tibetan wild boars [6,7]. Similarly, 11 candidate positively selected genes were found associated with a hypoxia response in ground tits [8]. Recently, several candidate genes were associated with high-altitude hypoxia in Tibetan sheep [9]. Almost all of the identified genes which belong to the list of 247 hypoxia genes that are priority candidates for adaptation to high-altitude hypoxia [12]; Our recent studies have demonstrated that the positively selected haplotypes of *EPAS1* was significantly associated with higher hemoglobin concentration for hypoxia adaptation of yak [13]. Hypoxia-inducible factors (HIFs) are transcription factors that respond to changes in the available oxygen in the cellular environment under high-altitude conditions. *EPAS1*, also known as *HIF-2α*, is a member of the HIF family that responds to changes in available oxygen in the cellular environment under high-altitude conditions. However, nature of the interaction between HIF-1α and aerobic metabolism is still incompletely understood [14], thus we hypothesized that additional factors likely mediate the fundamental metabolic events of high altitude adaptations in yak.

Matrix metalloproteinases 3 ( *MMP3*), also called stromelysin-1, belongs to the MMPs family that also includes gelatinases, collagenases, stromelysins, and membrane type matrix metalloproteinase (MT-MMP). MMPs were found initially to degrade extracellular matrix proteins, such as some types of collagens and proteoglycans [15]. Later, MMPs have been shown to proteolytically process growth factors, cytokines, and their receptors, leading to altered activities of these molecules [16–18]. In addition, it was reported that expressions of inducible nitric oxide synthase (*iNOS*) and *MMP3* are probably regulated by HIF-1α in the cellular response to hypoxic and inflammatory environments [19–21]. Therefore, inhibition or down-regulation of these molecules (or both) may exert anti-hypoxic and anti-inflammatory effects. Recent studies suggested that HIF-1α has two important regulators ADAM metallopeptidase domain 17 (*ADAM17*) and arsenite related gene 12 (*ARG12*) and one target gene *MMP3* [22]. The *ADAM17* and *ARG12* proteins affect HIF-1α stability and activity by regulating production of tumor necrosis factor α (TNF-α) and nitric oxide, respectively [23,24], whereas *MMP3* has key roles in numerous physiological processes [25]. However, whether *MMP3* gene plays a key role for the yak at high altitude remains to be elucidated.

In the present study, the aim was to identify the polymor phisms of *MMP3* in 4 yak breeds from different altitude and local cattle as a comparison to evaluate its association with high-altitude adaptation.

## MATERIALS AND METHODS

### Animals and DNA samples

All procedures involved in the handling and care of animals were approved by the Institutional Animal Care and Use Committee of Lanzhou Institute of Husbandry and Pharmaceutical Sciences, and all efforts were made to minimize suffering.

Four unrelated growing yak steers which are living at dif ferent altitudes were enrolled in present study including Pali yak (PL; n = 56), with a good adaptation and performance, its central production area is located at Pali town and Kangbu township of Rikaze district in Tibet of China [4], the average altitude is 4,300 m above sea level. Tianzhu white yak (TZ, n = 111) is well known for its distinct white coat cover, is a rare and special genetic resource that inhabits mainly the eastern end of Qilian Mountains and the northern edge of Qinghai-Tibetan Plateau (102°02′ to 103°29′E; 36°29′ to 37°41′N, 2,800 m) [4]. Datong yak (DY, n = 72) is the first artificially cultivated yak breed, created from a cross between a wild breed and a domestic breed in Datong of Qinghai Province (101°22′E; 37°15′ to 37°15′N, 3,100 m). Gannan yak (GY, n = 95) raising in the Gannan Tibetan Autonomous Prefecture of Gansu (100° 46′ to 104°45′E; 33°06′ to 35°43′N, 3,050 m). The various rangelands of the Plateau are characterized by their high altitude, very low annual average temperature (from −1°C to −5°C), short growing season (from June to September), and great seasonal variation in feed supply. For make the conclusion more convincing 25 local cattle samples were included in this study for a comparison.

Blood samples (10 mL) were obtained from the vena jugul aris with 2 mL acidic citrate dextrose for Genomic DNA extraction which were quickly frozen in liquid nitrogen and stored at −80°C. Genomic DNA was isolated using the commercially available Master Pure DNA Purification Kit (TIANGENE, Beijing, China) and the purity was assessed by spectrophotometer. Samples with an optical density ratio (260 nm/280 nm) between 1.7 and 1.9 were used for analysis.

### Primer design and polymerase chain reaction amplification

The final concentration of 100 ng/μL of DNA pools of 50 randomly selected individuals was subjected to the polymerase chain reaction (PCR). The partial fragments of *MMP3* were amplified by PCR with 6 sets of degenerated primers (Table 1), which were designed on the basis of available sequences of cattle (GenBank accession No. JH880344.1).

The 15-μL PCR solution contained 50 ng DNA template was carried out with 0.50 mM deoxyribonucleotide triphosphates, 3 mM MgCl_2_ and 0.75 U Taq DNA polymerase (TIANGENE, China). The PCR was performed using the following program: 94°C for 5 min, followed by 35 cycles of 94°C for 45 s, annealing at X °C (corresponding to Table 1) for 30 s, 72°C for 2 min; The final extension step was followed by a 10-min reaction at 72°C.

The PCR products were directly sequenced in both direc tions in an ABI 3730 DNA analyzer (Applied Biosystems, New York, USA). Sequences were analyzed using the DNASTAR 5.0 package (DNASTAR, Inc., Madison, WI, USA).

### Genotyping

High-resolution melting (HRM) analysis was performed for SNP genotyping and mutation scanning by the LightScanner platform (Idaho Technology Inc. Salt Lake City, UT, USA) with the designed primers of small fragment (Table 2). Plates were heated in the LightScanner from 55°C up to 95°C with a ramp rate of 0.10°C/s. The melting curve analysis was carried out using the LightScanner software package with CALL-IT software (Idaho Technology Inc., USA). Melting profiles were calibrated by internal oligonucleotide controls, and then normalized, grouped and displayed as fluorescence-versus-temperature plots or subtractive difference plots (-df/dt vs T).

### Statistical analysis

Statistical analyses were carried out with SPSS 19.0 software for Windows (SPSS Inc., Chicago, IL, USA). Allele and genotype frequencies among groups were compared using the chi-square test. The haplotypes and linkage disequilibrium for each pair of segregating sites and the exact p value of Hardy-Weinberg equilibrium (HWE) for multiple alleles were assessed using SHEsis [26].

## RESULTS

### Single-nucleotide polymorphism variations in *MMP3* gene

We successfully amplified the *MMP3* gene with those primers and the pooled DNA as the template, comparison between the sequence of yak *MMP3* (GenBank accession No. JH880344.1) and the pooled yak DNA samples revealed 2 SNPs. The two novel of SNPs rs2381 A→G, rs4331 C→G were found in intron V and in intron VII of *MMP3*, respectively.

### Frequency distribution of genotype and allele in *MMP3* gene

**Figure 1:**
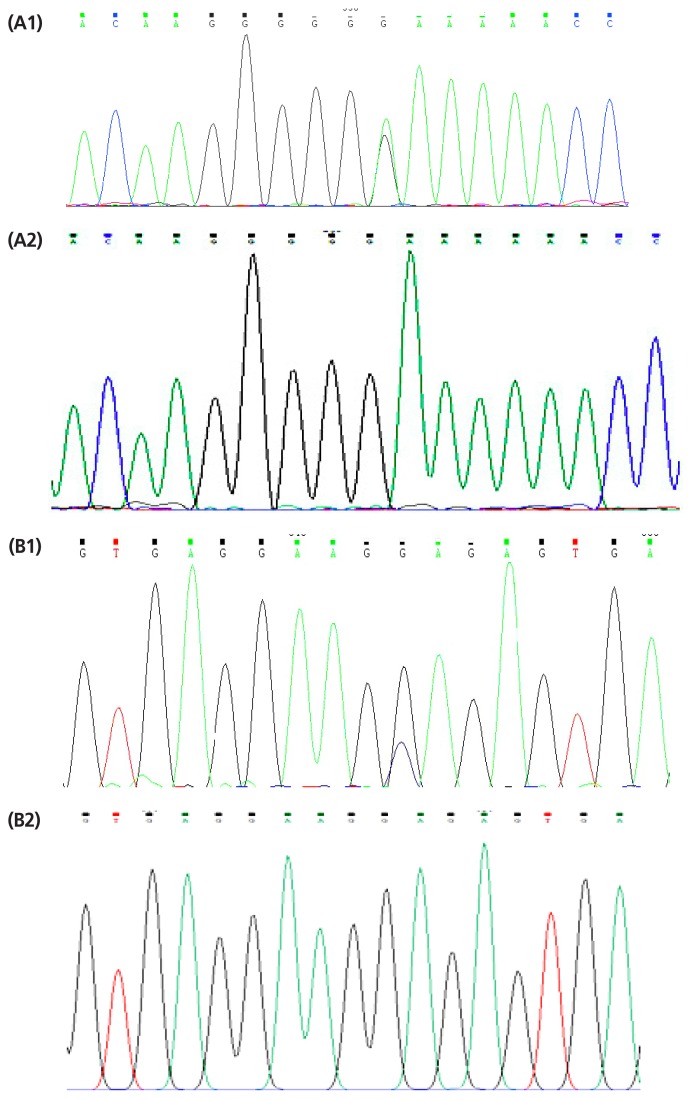
(A1) Sequencing maps at position of SNP rs2381 A→G in the yak *MMP3* gene. (A2) Sequencing maps at position of SNP rs2381 A→G in the cattle *MMP3* gene. (B1) Sequencing maps at position of SNP rs4331C→G in the yak *MMP3* gene. (B2) Sequencing maps at position of SNP rs4331C→G in the cattle *MMP3* gene. SNP, single-nucleotide polymorphisms; MMP3, matrix metalloproteinases-3.

SNP rs2381 A→G of *MMP3* was genotyped using HRM. The result identified three genotypes (GG, GA, AA; Figure 1A); GG genotype and G allele were the dominant among the four yak breeds. PL, DT, TZ of rs2381 A→G were in HWE (p>0.05), while GN yaks of rs2381 A→G were in HWE (p<0.05) (Table 3). Three genotypes (CC, CG, GG; Figure 1B) were also selected for the SNP rs4331 C→G, CC genotype and C allele were the dominant among the four yak breeds. The results suggested that PL and DT yaks of rs4331 C→G were in HWE (p>0.05), while GN and TZ yak of rs4331 C→G were in HWE (p<0.05) (Table 4). However, SNP rs2381 A→G and rs4331 C→were specific to yak, cattle were homozygous with G allele at the same locus (Figure 1 A2, B2).

Comparison analysis showed that differences in genotype and allele frequencies among the 4 yak breeds, showed that rs2381 A→G mutation, high altitude PL yak with middle-altitude GN, DT, and low-altitude TZ have significant differences between genotype frequencies or Allele frequencies, and other breeds were not significant (Table 5).

### Haplotype analysis

Four haplotypes (AC, AG, GC, and GG) were found in the 4 yak breeds, haplotype GC yaks population was significantly higher than in others. The result showed haplotype GC was the dominant among the 4 yak breeds (Table 6). Compared with that Pearson’s p value in different breeds, between PL yak (high-altitude) with GN, DT yaks (middle-altitude) and TZ yaks (low-altitude) were significant differences (Table 7).

## DISCUSSION

Vertebrates at high altitude are subjected to hypoxic conditions that challenge aerobic metabolism. Yaks are a hypoxia-tolerant species that live in an extremely inhospitable high-altitude environment, which has high UV radiation and a low partial pressure of oxygen compared with low-altitude areas [4,27]. Understanding how yaks cope with the combined effects of hypoxia and cold can provide important insights into the process of adaptive evolution.

It was reported that expressions of *iNOS* and *MMP3* are probably regulated by *HIF-1α* in the cellular response to hypoxic and inflammatory environments [19–21]. However, research focusing on the functional roles of *MMP3* involved in hypoxic responses on the yak, has been limited until now. In the present study, the SNPs genotyping and mutation scanning at the *MMP3* locus were selected in total of 344 individuals from 4 domestic Chinese yak breeds resident at different high altitude using HRM and DNA sequencing techniques. To the best of our knowledge, this is the first study to characterize polymorphisms in the target gene (*MMP3*) of HIF for hypoxia adaptation in yak.

Significant differences in the allele and genotype frequen cies of the rs2381 A→G polymorphism among the four yak breeds were detected. Previous researches demonstrated that yaks, compared to indigenous cattle (*Bos taurus*), have evolved specific adaptation mechanisms in physiology, nutrient and highly efficient energy metabolism [5,28–30]. However, the underlying mechanisms of this highly coordinated metabolic response are only beginning to be understood at the molecular level. HIF-1α, a master regulator of the cellular hypoxic response has been shown to control mitochondrial function [31] and is essential for this repression of mitochondrial respiration [32] during hypoxia. Our Recent studies have also demonstrated that the positively selected haplotypes of EPAS1 was significantly associated with the higher haemoglobin (Hb) content of Tibetan yak [13]. The result was opposite from the mechanisms underlying plateau adaptability of identifying candidate genes for genetic adaptation to the Tibetan Plateau for Tibetan people, which demonstrated that the positively selected haplotypes of *EGLN1* and *PPARA* were significantly associated with the low haemoglobin content of Tibetan people [12]. However, data from our physiological characteristics of several species yaks have indicated that modifications of Hb function is not totally conclusive on the yak adaptation at high altitude, the physiological adaptation was achieved in yaks maybe by increase in RBC and blood oxygen affinity, decrease in mean cell volume, in addition enhanced enzymatic activity play a key role in more added metabolic cost and anaerobic metabolism [5]. Therefore, here we speculate that high-altitude yaks have better adaptability in a hypoxic environment.

The results of HRM genotyping showed that both of rs2381 A→G and rs4331 C→G in Gannan yak populations were in Hardy-Weinberg disequilibrium (p<0.05), the result showed in the site may suffered from a larger selection pressure in the GN yak breeds. In addition, SNP rs2381 A→G was specific to yak, cattle were homozygous with G allele at the same locus (Figure A2, B2). The significant difference among the four populations may be caused by the differences in the breeds, but the significant differences (p<0.05) in allele or genotype frequencies of SNP rs2381 A→G in PL yak (high altitude) and GN, DT yak (middle-altitude), TZ yak (low-altitude) (p<0.05), which have lived at high altitude for many generations, is likely the result of a long period of natural selection for environmental adaptation [33]. However, no significant difference in allele or genotype frequencies of SNP rs4331 C→G among in 4 yak breeds (p>0.05), and we suspect that the differentiation between high-altitude and low-altitude yaks is relatively young because some mutations with relevance to high-altitude adaptation are not spread completely throughout the yak population. The 2 SNPs were in moderate linkage disequilibrium in high-altitude yaks (PL) but not in middle-altitude (GN, DT) and low-altitude (TZ) yaks, which indicated that *MMP3* may be a rarely occurring recombination in these 4 yaks and selection is likely a reason for the moderate linkage disequilibrium (Table 8).

Tissue hypoxia can reportedly up-regulate a series of local factors that contribute to angiogenesis and the growth of new capillary vessels, which increase delivery of both oxygen and energy substrates such as glucose. Clearly *MMP3* expression is induced by hypoxia, and proteolytically process growth factors, cytokines, and their receptors, leading to altered activities of these molecules [16–18]. It is supposed that the 5A/6A polymorphism may be associated with the *MMP3* gene promoter activity under interleukin and tumor necrosis factor α (TNF α) stimulation and it may influence the transcription of the gene through the regulation by cytokines released by tumor cells [17,18]. It is possible that the 5A/6A polymorphism of the MMP3 gene may not be directly associated with development of ovarian cancer [23]. These findings illustrated the G to A substitution of in MMP3 provides better protection against hypoxia. These yaks inhabit an area of the plateau with a hypoxic environment. The better hypoxia adaptation of A allele carriers and hypoxic environment selection over a long period might result in the higher frequency of the A allele in the high-altitude yak population.

Study of haplotypes has proved useful for a more compre hensive analysis of association results. Haplotype-based analysis showed significant differences in the haplotype distribution among the 4 yak breeds (p<0.01) especially the haplotypes GC. These results suggested that haplotypes GC constructed by SNP rs2381 A→G may impart better adaptation capability in the hypoxic environment of the plateau.

Take together, our results indicate that *MMP3* may have been subjected to positive selection in yak, especially that and the SNP (rs2381 A→G) mutation and GC haplotypes might be advantageous for yak adaptation to high-altitude environments. These data will help to elucidating the biological roles of *MMP-3* and decoding the mechanisms of mediated gene regulation of hypoxia in domesticated animal model.

## Figures and Tables

**Table 1:** Primes used for polymerase chain reaction amplification

Primers	Primer sequences (5′-3′)	Tm (°C)	Length (bp)	Locus (bp)
P-1	F:5′ CTTCTTTTCTCAATCCCACA 3′ R:5′ ACTCTGCCTTTACACTTCGT 3′	56.8	607	621–1,228
P-2	F:5′CAAAACCACCTTAGTAGCAG 3′ R:5′ AATGGCAGAATCAACAGC 3′	56.8	936	1,335–2,271
P-3/4	F:5′ TACGCAAGCCCCGATGT 3′ R:5′ AAAGGCGGAACCGAGTG 3′	56.8	1127	2,022–3,149
P-5	F:5′ GGGAGTAATTGGATATGGC 3′ R:5′ GGAAAGTGGAGCGTCAG 3′	45.5	920	2,752–3,672
P-6	F:5′ GTTCTCCCTATCTCCATCC 3′ R:5′ ACTTCACTTTCACGCATTG 3′	56.8	1332	3,961–5,293
P-7/8	F:5′ TACATTGGCAGGAGGATT 3′ R:5′ AGGTGGGATGGAGAAGC 3′	54.5	761	5,687–6,448

**Table 2:** Information of primer sequences for genotyping mutations by high-resolution melting

Gene	Loci	Primer sequences (5′-3′)	Tm (°C)	Length (bp)
*MMP3*	rs2381	F: ATCATATTTGCAGTTAGAGGTAA R: CTAATGAAACAACACTAGATAAAA	61.8	83
	rs4331	F: TCCTAGAGGATGTGGAAATGGAG R: TCTTCCGCCTTTCAGCATA	66.3	73

*MMP3*, matrix metalloproteinases-3.

**Table 3:** Allele and genotype distribution of single-nucleotide polymorphism rs2381 A→G in the four yak breeds

Breeds	Genotypes (%)			Alleles (%)		Hardy-Weinberg χ^2^
	
	GG	GA	AA	G	A	
PL (n = 56)	55 (0.982)	0 (0.000)	1 (0.018)	110 (0.982)	2 (0.018)	7.57e-014
GN (n = 94)	68 (0.723)	20 (0.213)	6 (0.064)	156 (0.830)	32 (0.170)	0.016743
DT (n = 96)	75 (0.781)	14 (0.146)	7 (0.073)	164 (0.854)	28 (0.146)	4.91e-005
TZ (n = 94)	78 (0.830)	10 (0.106)	6 (0.064)	166 (0.883)	22 (0.117)	2.59e-006

PL, Pali; GN, Ganan; DT, Datong; TZ, Tianzhu.

**Table 4:** Allele and genotype distribution of single-nucleotide polymorphism rs4331 C→G in the four yak breeds

Breeds	Genotypes (%)			Alleles (%)		Hardy-Weinberg χ^2^
	
	CC	CG	GG	C	G	
PL (n = 56)	43(0.768)	7(0.125)	6(0.107)	93(0.830)	19(0.170)	3.18e-005
GN (n = 95)	67(0.705)	21(0.221)	7(0.074)	155(0.816)	35(0.184)	0.009951
DT (n = 96)	60(0.625)	20(0.208)	16(0.167)	140(0.729)	52(0.271)	3.72e-006
TZ (n = 92)	59(0.641)	21(0.228)	12(0.130)	139(0.755)	45(0.245)	0.000248

PL, Pali; GN, Ganan; DT, Datong; TZ, Tianzhu.

**Table 5:** Comparison of differences in genotype and allele frequencies

Single-nucleotide polymorphism	Allele genotype					

	PL-GN	PL-DT	PL-TZ	GN-DT	GN-TZ	DT-TZ
rs2381	5.72e-005	0.000310**	0.002206**	0.514664	0.141410	0.406135
	0.000279**	0.002641**	0.014512*	0.482509	0.134105	0.676567
rs4331	0.749641	0.044286*	0.128856	0.043561*	0.154713	0.560615
	0.301108	0.190738	0.231519	0.140378	0.411467	0.771302

PL, Pali; GN, Ganan; DT, Datong; TZ, Tianzhu.

**Table 6:** Haplotype distribution of single-nucleotide polymorphisms (rs2381 A→G, rs4331 C→G) in the three yak breeds

Haplotype	PL (%)	GN (%)	DT (%)	TZ (%)
AC	0.9	13.0	8.7	7.8
AG	0.9	4.2	5.9	2.2
GC	82.1	68.2	64.2	67.7
GG	16.1	14.6	21.2	22.3

PL, Pali; GN, Ganan; DT, Datong; TZ, Tianzhu.

**Table 7:** The haplotype Pearson’s p value of single-nucleotide polymorphisms in the four yak breeds

Single-nucleotide polymorphism	PL-GN	PL-DT	PL-TZ	GN-DT	GN-TZ	DT-TZ
Pearson’s p value	0.000826**	0.001183**	0.007945**	0.197437	0.092621	0.325865

PL, Pali; GN, Ganan; DT, Datong; TZ, Tianzhu.

**Table 8:** Analysis of linkage disequilibrium of the two polymorphisms in the four yak breeds

Breeds	D′	r^2^
PL (rs2381, rs4331)	0.398	0.014
GN (rs2381, rs4331)	0.068	0.004
DT (rs2381, rs4331)	0.184	0.016
TZ (rs2381, rs4331)	0.110	0.000

PL, Pali; GN, Ganan; DT, Datong; TZ, Tianzhu.
